# Residual Mechanical Properties of Concrete Made with Crushed Clay Bricks and Roof Tiles Aggregate after Exposure to High Temperatures

**DOI:** 10.3390/ma9040295

**Published:** 2016-04-19

**Authors:** Ivana Miličević, Nina Štirmer, Ivana Banjad Pečur

**Affiliations:** 1 Department for Materials and Structures, Faculty of Civil Engineering, University of Osijek, Osijek 31000, Croatia; 2 Department for Materials, Faculty of Civil Engineering, University of Zagreb, Zagreb 10000, Croatia; ninab@grad.hr (N.Š.); banjadi@grad.hr (B.P.)

**Keywords:** concrete, crushed brick, crushed tile, ultrasonic pulse velocity, mechanical properties, high temperatures

## Abstract

This paper presents the residual mechanical properties of concrete made with crushed bricks and clay roof tile aggregates after exposure to high temperatures. One referent mixture and eight mixtures with different percentages of replacement of natural aggregate by crushed bricks and roof tiles are experimentally tested. The properties of the concrete were measured before and after exposure to 200, 400, 600 and 800 °C. In order to evaluate the basic residual mechanical properties of concrete with crushed bricks and roof tiles after exposure to high temperatures, ultrasonic pulse velocity is used as a non-destructive test method and the results are compared with those of a destructive method for validation. The mixture with the highest percentage of replacement of natural aggregate by crushed brick and roof tile aggregate has the best physical, mechanical, and thermal properties for application of such concrete in precast concrete elements exposed to high temperatures.

## 1. Introduction

The production of mineral raw materials in the European Union member states is very dynamic because it is fairly dependent on the realization of and investment in projects approved for a defined period of time [1]. Aggregate production in the 15 European Union countries comprising the members of the European Aggregates Association (UEPG) amounted to 2620 million tonnes of sand, gravel and crushed stone in 2000, which represents a production of 6.9 tonnes per capita [1]. It is obvious that this level of annual aggregate production has a significant impact on the environment. It is our responsibility to reduce consumption of natural mineral resources and to increase the use of secondary raw materials, *i.e.*, recycled materials. Aggregate production is economically and socially important as well: the value of natural raw materials produced annually amounts to approximately 35 billion euros, and the aggregate production industry employs around 250,000 people. The estimated demolition waste material in the EU member states amounts to approximately 180 million tons per annum, which is 1.3 kg a day per capita. There are significant differences in produced construction waste quantities within the European Union; for instance, Germany and the Netherlands produce about 1.9 kg per capita daily, whereas Sweden, Greece and Ireland produce less than 0.5 kg [1].

More than 80% of the total construction obtained by waste in certain European countries, such as The Netherlands, Belgium and Denmark, is recycled [2]. In this way, these highly developed countries simultaneously decrease the need for new exploitation of pristine natural areas and resolve the problems of construction waste, which is otherwise often disposed of in landfills. Since the usage of recycled materials in all areas of human activity is a global trend, which is true for the construction sector as well, it is now necessary to use recycled construction waste in the production process of concrete within Croatian borders.

Crushed clay bricks and roof tiles are among the best aggregates, for use in concrete are known have to resistance at high temperatures, and concrete with such aggregates performs much better than similar concretes containing granite aggregate [3]. Clay bricks and roof tile aggregates are thermally stable. This is probably why they perform well in concrete subjected to high temperatures. The resistance of clay bricks and roof tiles to high temperatures is an important characteristic: it has been recognized that brickwork masonry and clay roofing are very effective in resisting and preventing the spread of fire. When brick and roof tile materials are used as aggregates in concrete, they are not expected to negatively affect the resistance to high temperatures. To ensure the resistance to high temperatures, crushed brick and roof tile aggregate concrete should be kept dry. When they are wet, the internal steam pressure that is created at high temperatures can cause spalling [4]. The low thermal conductivity of crushed clay brick and roof tile aggregate concrete also improves its resistance to high temperatures. Such concrete is much better protected against early heating, as it keeps its structural integrity under high temperatures for a longer period than ordinary concrete.

## 2. Experimental Investigation

### Materials and Methods

Crushed brick (CB), crushed roof tile (CT) and dolomite/limestone (natural aggregate—NA) have been used in this study as aggregates for concrete mixtures. There was no need for additional processing of dolomite because it already was separated into fractions. The bricks and roof tiles were crushed at the brick and roof tile factories and delivered to the concrete-mixing location. Since both CB and CT represent waste products in the process of making new bricks and roof tiles, no additional processing of these materials was required. If CB and CT are to be used as aggregates for concrete, it is necessary to prove that they are adequate replacements for conventional NA. The CB and CT were sieved into 0–4 mm, 4–8 mm and 8–16 mm fractions. Based on the granulometric composition, particle size groups were determined as shown in Table 1. The particle shape of the coarse aggregates was determined as a Shape Index in accordance with [5], and it was selected in accordance with HRN EN 933-4 [6]. The Concrete Constructions Technical Regulations (TPBK) indicate the highest allowed particle size for concrete constructions, which is SI_20_ [7]. Table 1 shows that all three aggregate types comply with the mandatory regulations: the fine particle content is determined in accordance with HRN EN 933-8 [8] and the TPBK. Particle size in relation to fine particle percentage is determined in accordance with [5]. The largest acceptable values of the fine particle size prescribed by the TPBK are: *f*_3_ for natural fine aggregate, *f*_10_ for crushed and mixed fine aggregates, and *f*_1,5_ for the coarse fraction. Based on the listed limits, as shown in Table 1, all three aggregates comply with the mandatory regulations. The bulk density was tested in accordance with standard HRN EN 1097-3 [9]. The TPBK dictate that the aggregate bulk density must comply with the project terms or customer demands. Specific density and water absorption were tested in accordance with standard HRN EN 1097-6 [10]. Like for the bulk density, the TPBK dictates that the specific density of aggregate particles must comply with the project terms or the customer demands. The data in Table 1 indicate that CB and CT have a lower density than NA. It was expected, then, that concrete mixtures with CB and CT will have lower density values depending on the replacement percentage. Table 1 shows that the water absorption of CB and CT is significantly higher than for dolomite. It is necessary to take into account the water absorption when designing the concrete mixture composition, since the values are high: 16%–19% for bricks and 9%–11% for roof tiles, depending on the fraction.

The coarse aggregate abrasion test (Los Angeles–LA) was conducted in accordance with the standard HRN EN 1097-2/A1 [11] and the TPBK. It is evident from the results in Table 2 that all three aggregate types comply with the mandatory regulations and that the brick and roof tile waste show satisfactory results, despite the expectations of a reduced abrasion resistance.

Aggregate freezing and thaw resistance were tested in accordance with standard HRN EN 1367-2 [12], by which a magnesium sulphate test was also conducted. The particle size distribution that aggregates need to comply with depends on the end use of the concrete and was determined by standard [5] and the TPBK. Based on the results of the conducted tests shown in Table 1, it can be concluded that all three aggregate types comply with the mandatory regulations and are all in the same magnesium sulfate class.

**Figure 1:**
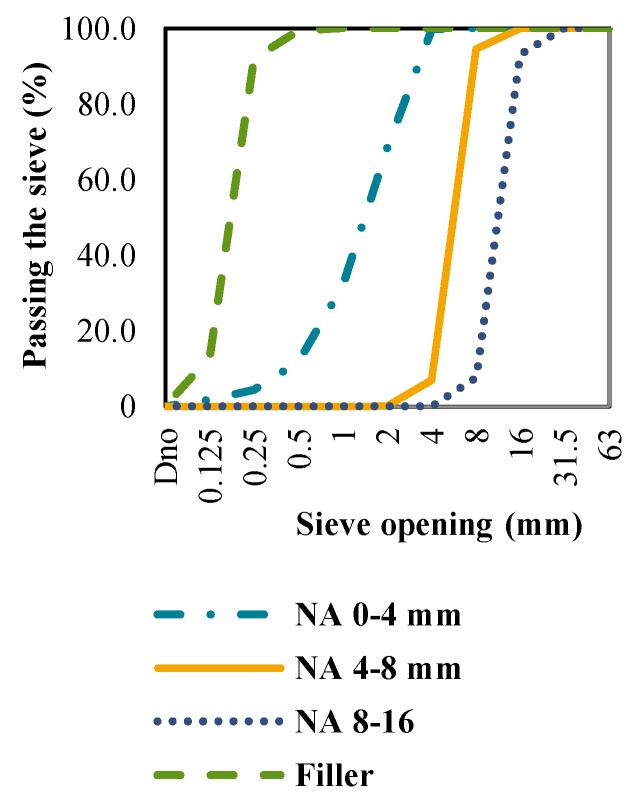
Grading curves for dolomite.

**Figure 2:**
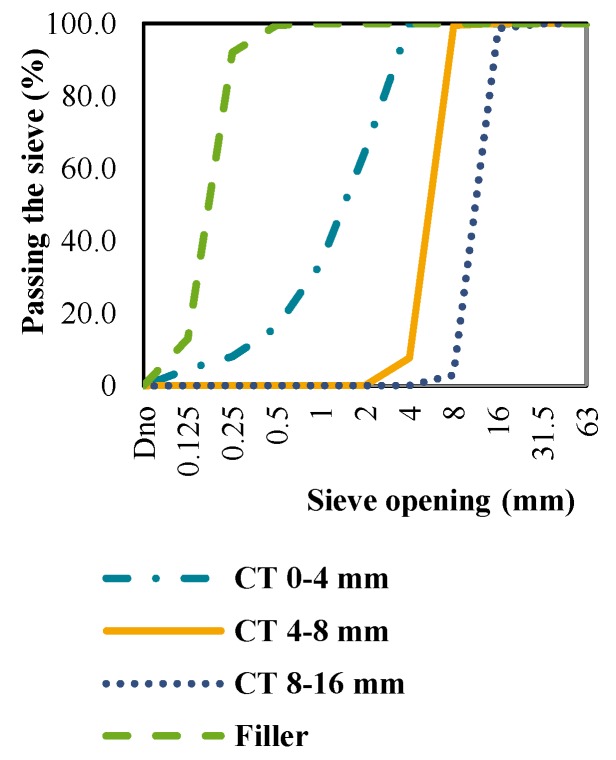
Grading curves of crushed roof tiles.

**Figure 3:**
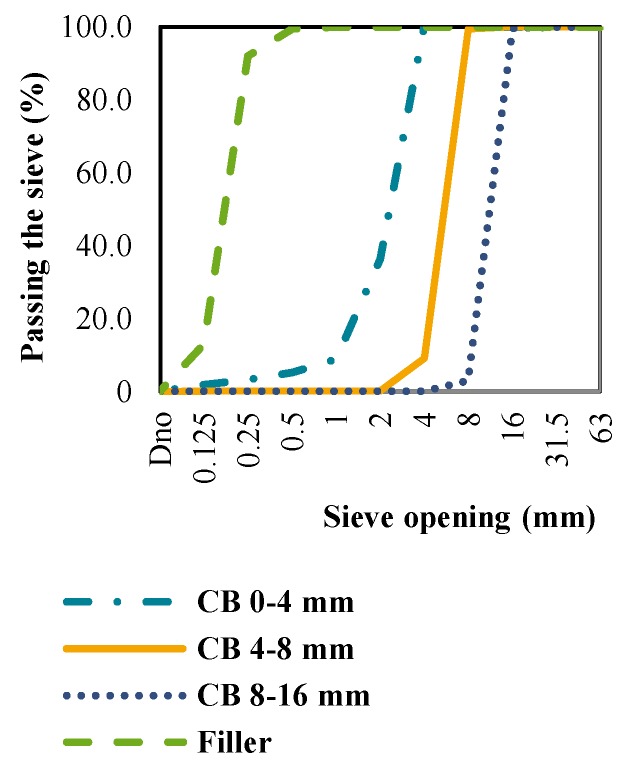
Grading curves of crushed bricks.

The grading curves of CB, CT and NA in further tests are shown in Figure 1, Figure 2 and Figure 3. The curves represent the mean values of three siftings of an individual aggregate type. Based on the grading composition, the aggregate classes were determined as shown in Table 1. The table shows that the CB and CT satisfy the mandatory conditions of standard HRN EN 12620 [5] and the Concrete Constructions Technical Regulations (TPBK) [7], and can therefore be used to replace conventional NA.

The mixture compositions are shown in Table 2. Crushed aggregate has been mixed with the water before placing it in the container. After the aggregate has reached the saturated dry-surface state, it was stored in the container to stay in this condition until the usage. The container was closed hermetically in order to prevent the loss of humidity. The aggregates were stored for 24 h under these conditions before the beginning of concrete mixing in accordance with previous research [3,4], in which it was determined that 24 h are sufficient to reach saturated dry-surface state. When mixing the components, the aggregates were in a saturated, dry-surface state. The total amount of water added to the mixture is shown in Table 2. NA powder was used as a filler. By analyzing the test results for CB and CT aggregates, it was possible to conclude the following:

1. CB and CT can be used as full or partial replacement for NA (in this study dolomite aggregates).
2. Special attention must be paid to the concrete composition with regard to the water quantity required and the percentage of replacement of NA by brick and roof tile waste, depending on concrete requirements.

Portland cement CEM I 42.5 N was used throughout the preliminary experimental test phase. Its characteristic mechanical, physical and chemical properties are in compliance with standard HRN EN 197-1 [13]. Polycarboxylate based superplasticizer Sika ViscoCrete (20 Gold) was added to obtain a high workability and early strength development; it is a brown, water-soluble fluid. The characteristics of the superplasticizer were tested by the manufacturer.

Concrete was mixed in a laboratory mixer of 70 L maximum volume. Each of the nine compositions was mixed three times in order to determine the repeatability of the test results. Concrete was consolidated on a vibrating table with 150 Hz frequency.

## 3. Results and Discussion

### 3.1. Properties of Fresh Concrete

After the concretes (Table 3) were mixed, the fresh-state properties were tested, as reported in Table 3. Tests were conducted in accordance with the HRN EN 12350 [14] group of standards for fresh concrete. Each test was repeated three times for each individual mixture, and the mean values are shown in Table 3.

By comparing the mixtures in Table 3, the following can be concluded:

1. As expected, the densities of mixtures containing CB and CB aggregates are lower than the density of the reference mixture with NA.
2. The consistency of all nine mixtures is in accordance with the recommendations of the standard HRN EN 206-1 [15].
3. The air content is lower in the RM mixture (2.53%) than in the mixtures with CB and CT (4% to 5.5%), which was expected due to the more irregular particle shape of coarse aggregates.

### 3.2. Properties of Hardened Concrete at a Temperature of 20 ± 5 °C

Concrete mixtures were cast in molds and the samples were covered with a plastic sheet; they were then left in molds for 24 h. After they were demolded, the concrete samples were placed in a water tank in a digitally controlled temperature environment of 20 °C until they were tested. Table 4 and Table 5 show the results of the hardened concrete properties at 28 and 56 days. The tests were conducted with three samples of an individual mixture and the mean values are given in Table 4 and Table 5.

The density of CB and CT aggregate concrete is lower than that of the reference mixture, as was expected. Any further increase of the CB and CT percentage may lead to the production of lightweight concretes.

Table 4 and Table 5 show that the compressive strengths of all nine concrete mixtures comply with the requirements of standards HRN EN 15037-1 [16] and HRN EN 15037-2 [17], which define the minimum concrete classes C12/15 for load-bearing floor blocks and C20/25 for precast-concrete load-bearing beams. The compressive strengths of the 28-day-old mixtures with CB and CT aggregates are on average 28% lower than that of the RM, as expected. The compressive strengths are comparable with conventional concrete containing NA and were achieved for concrete with CB and CT as aggregates, taking into account the 45% limit for coarse and 50% limit for fine particle fractions in substitution of NA with CB and CT.

The flexural strength of mixtures with CB and CT aggregates is on average 9% lower than that of the NA concrete mixture.

The gas permeability of concrete was measured on cylinder-shaped samples, 50 mm in diameter and 50 mm in length. All tests were conducted on three samples and Table 5 shows the mean values. The test results show that each of the nine concrete mixtures falls into the category of poor-quality concrete with regard to gas permeability. This can be explained by the preparation of the samples in plastic tubes, 50 mm in diameter, wherein it was not possible to properly consolidate the concrete that caused the presence of cavities. The tests should be repeated on 10 cm samples in order to validate the results. The results also indicate that concretes with crushed bricks and roof tiles have a higher gas permeability than traditional concrete. It is presumed that the reason for the higher gas permeability is the particle shape of CB and CT, which is sharper and the particles are more porous than dolomite grains.

According to Table 5, the statistical modulus of elasticity of concrete mixtures with CB and CT aggregates is up to 50% lower than that of concrete with NA. When designing load-bearing construction elements using concrete with CB and CT as aggregate, for which shrinkage is relevant, it is important to take into consideration the lower modulus of elasticity. A lower modulus of elasticity reduces the stiffness of the construction. Since the stiffness is lower, the use of concrete with CB and CT is recommended for example in vertical elements as filling material (for instance, in an infilled frame). It is also suggested that concrete blocks with such CB- and CT-infilled frames could provide a better earthquake resistance. The use of such concrete in horizontal construction elements (floor beams, floor slabs, *etc.*) would result in elements with a higher drying shrinkage contribution at identical strain when compared to concrete with NA. However, in short elements such as floor blocks, the modulus of elasticity plays no significant role, and the use of CB and CT in resisting and semi-resisting floor blocks may therefore be considered possible.

By comparison with the ultrasonic speed velocity results listed in Table 4 and Table 5, it can be concluded that CB and CT samples have a lower ultrasonic speed velocity, which may indicate the appearance of defects and cracks, a higher porosity and other non-homogeneities. Since all mixtures have ultrasonic speed velocity above 3.60 km/s, it may be concluded that they are in the high-quality concrete class [18].

**Figure 4:**
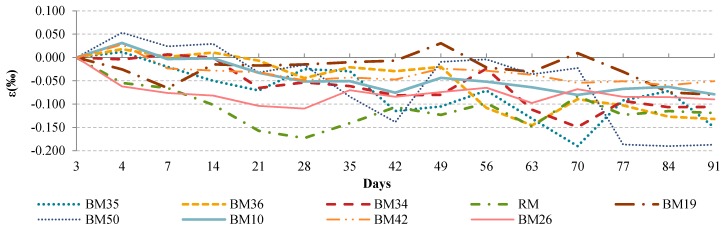
Comparison of drying shrinkage of concrete mixtures.

The drying shrinkage of concrete, as shown in Figure 4, was measured with samples constantly exposed to humidity conditions of 95% ± 5% relative humidity. Like for all other hardened-concrete properties, drying shrinkage was tested with three samples of the same mixture. Drying shrinkage of each sample was measured in two different measuring lines facing each other on days three, four, seven, and afterwards every seven days until the 91st day, which is 15 times in total.

Figure 4 shows a comparison of drying shrinkage measured for 91-day-old mixtures. Reason for the inconsistent trend could be the relatively high percentage range of relative humidity, and this issue will be addressed in future research. Despite inconsistent trends for particular concrete mixtures, the results indicate that the lowest level of drying shrinkage was observed for samples with CB and CT. This implies a positive effect for the final use of these kinds of aggregates in precast concrete elements.

### 3.3. Hardened Properties of Concrete after Exposure to High Temperatures

**Figure 5:**
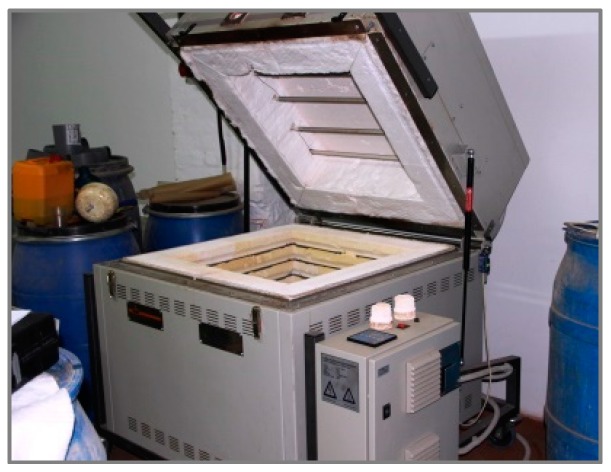
Electric furnace.

**Figure 6:**
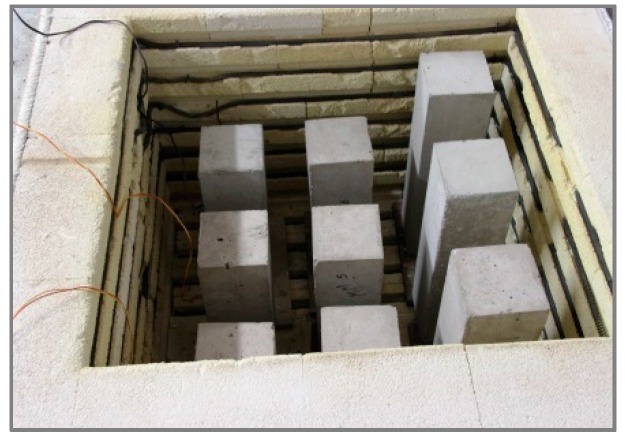
Samples in furnace.

**Figure 7:**
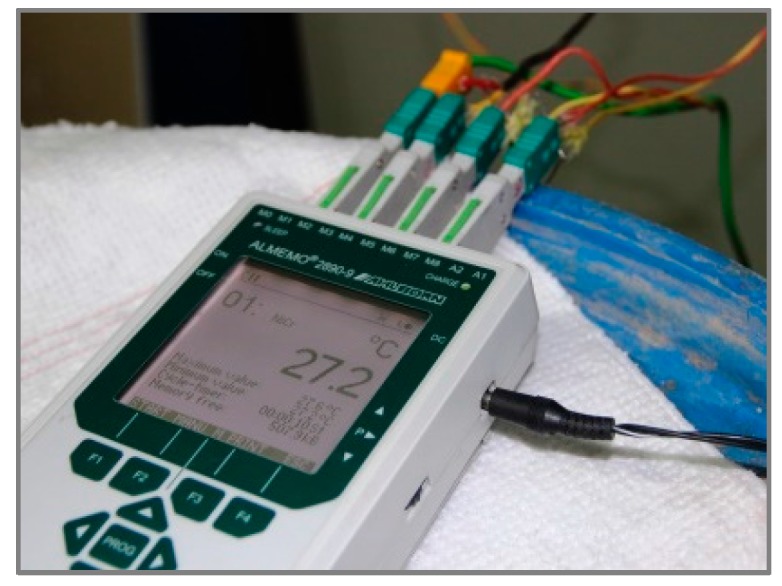
Measurement of the temperature in samples.

Samples for testing mixture properties after exposure to high temperatures were made using identical mixture compositions, and the samples were cured in an identical manner up until 28 days of age compared to samples tested at room temperature. After 28 days, before their placement in an electric furnace at 105 °C, the samples were kept at room temperature for seven days in order to avoid the emergence of cracks due to the sudden drying at 105 °C. The drying procedure was carried out until the samples were dried completely. In order to be able to design concrete structures as well as to assess the safety with regard to thermal events or after a real accident according to [19], it is necessary to have detailed knowledge of the concrete properties for a temperature range from 20 °C to at least 750 °C. In some cases, the data of concrete properties are needed up to 1000 °C. The samples were then exposed to high temperatures in the electric furnace. Prior to exposure in the electric furnace, the weight of samples was determined to check humidity in the period between the drying and high temperature exposure. If the weight increase was higher than 1% in comparison to dry weight, the samples were returned to drying procedure as described above. The temperature rate in the electric furnace was 1 °C/min and was measured via a thermo probe located on the furnace and a control probe placed in the furnace. The heating regime was in accordance with the recommendations of RILEM TC 200-HTC [19]. Thermocouples integrated into the three samples of each mixture enabled the register temperature rise in the individual samples (Figure 5, Figure 6 and Figure 7). Based on previous research of the authors and based on recomendations RILEM TC 200-HTC, [19] to obtain reproductible results, specimens were at identical curing conditions after exposure to high temperatures. Specimens were cured at a temperature of 20 ± 5 °C and in relative humidity 50% ± 5%, under conditions without any moisture exchange, for seven days after which testing of mechanical properties took place.

**Figure 8:**
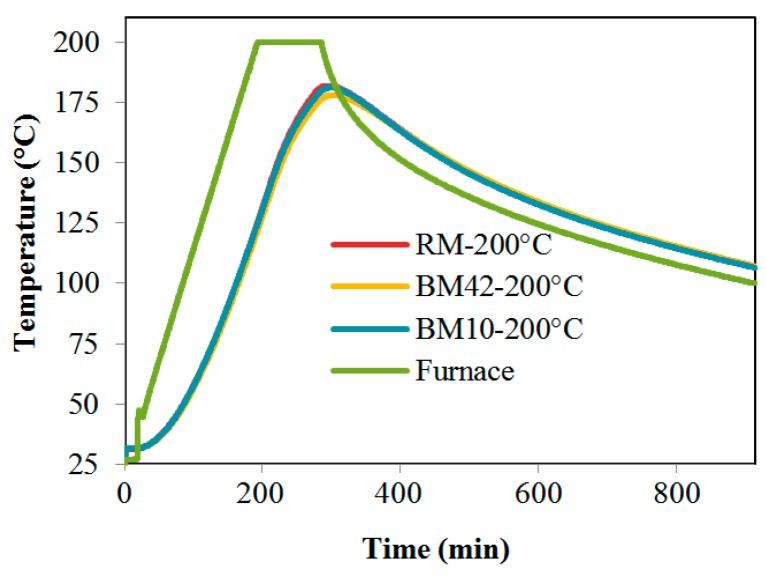
Heating regime of mixtures RM, BM10 and BM42 for 200 °C.

**Figure 9:**
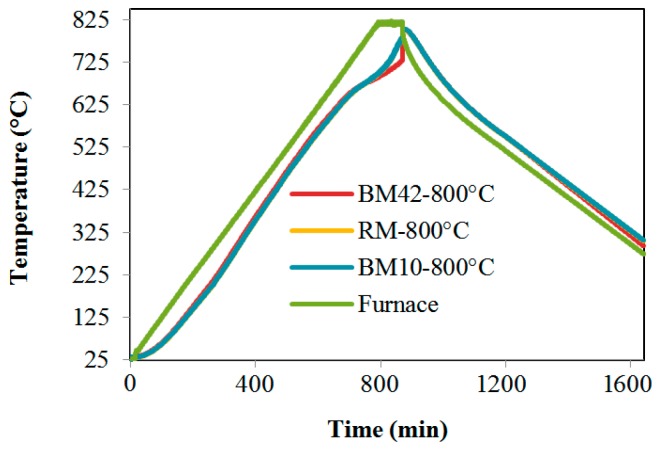
Heating regime of mixtures RM, BM10 and BM42 for 800 °C.

The heating and cooling regimes of samples exposed to temperatures of 200 °C and 800 °C are shown in Figure 8 and Figure 9. Figure 8 indicates that, during the heating of samples at 200 °C, the temperature rise in all three mixtures was approximately the same. During exposure to a temperature of 800 °C, a higher temperature rise is observed in the RM samples, as shown in Figure 9. The reason for this elevated temperature rise in the RM samples may be found in the higher thermal conductivity of RM concrete when compared to concretes with CB and CT, as was confirmed by a thermal conductivity coefficient test carried out for concrete in [20]. Figure 8 also shows a lower temperature rise in samples with CB and CT as aggregates, which may imply that these concretes have a higher heat conductivity when compared to concrete with NA. 

Concrete, like the majority of materials, changes mass under the influence of temperature. The weight was measured before and after high temperature exposure on digital scales with a precision of ±0.1 g.

**Figure 10:**
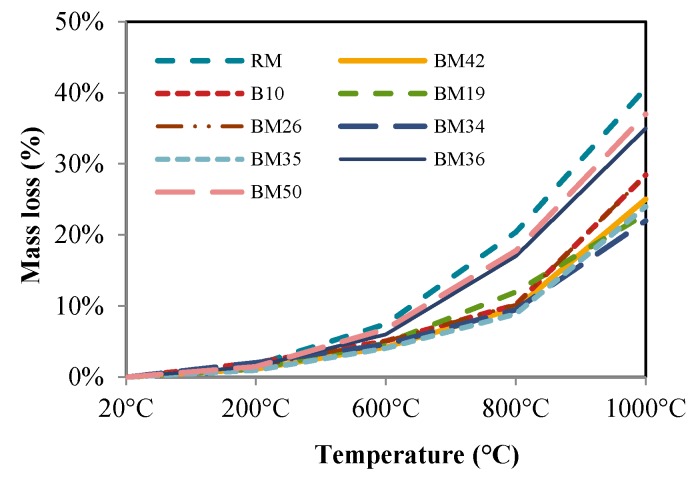
Mass loss after exposure to high temperatures.

Mass losses after heating are shown in Figure 10. The residual mass, the percentage of the remaining mass in comparison to the initial mass before heating, was approximately 98% for all samples exposed to 200 °C. After the exposure of samples to temperatures of 600, 800 and 1000 °C, the RM lost significant mass as opposed to its initial mass, whereas the mixtures with CB and CT aggregates displayed a better resistance to mass loss due to heating.

The concrete mass loss after heating was calculated using Equation (1) (illustrated in Figure 10):

(1) $$M_{loss}=\left(M_{before}-M_{after}\right)/M_{before}$$

It is also observable that the lowest mass loss was obtained for the mixtures with the highest percentage of CB and CT aggregates. The mass loss in concrete with CB and CT aggregates is about 25% after exposure to 1000 °C, while the referent mixture mass loss is 40.6%. This indicates that it is possible to achieve a significantly lower concrete mass loss during high temperature exposure when replacing 50% of fine particles and 75% of coarse particles of NA with CB and CT. Since all samples had a weight increase of up to 1% before heating, the mass loss presumably originates from water evaporation. It is presumed that this results in a lower pressure, causing less concrete deterioration, but it may also result in better integrative properties of concrete components, *i.e.*, higher residual mechanical properties.

**Figure 11:**
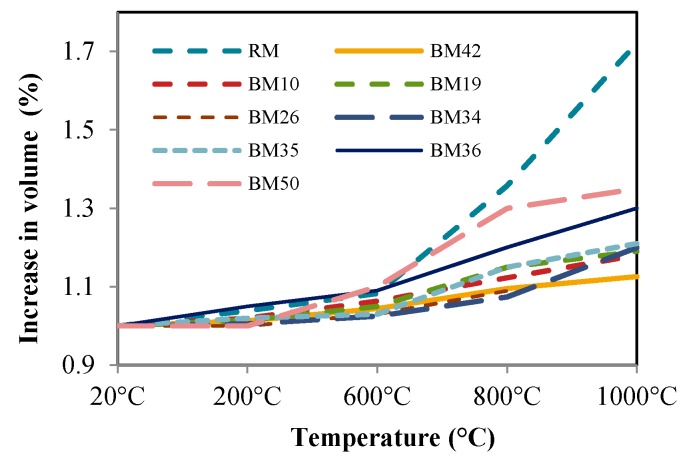
Relative volume as a function of high temperatures.

Concrete changes its volume when exposed to different temperatures. Therefore, the volume of each sample was measured before and after exposure to high temperatures. Figure 11 shows the percentage of sample volume increase after exposure to high temperatures. The results indicate that the least observable change in volume was noted for BM36 samples. For example, the sample volume increase after exposure to 800 °C for RM was 36%, whereas, for BM36, it was 9.5% and for B10 12.3%. These results indicate that concrete with CB and CT as aggregates have an approximately 25% less volume change due to high temperature exposure.

Since a fire causes stress in a construction, the resulting stresses are higher for mixture RM. If the space for the change of length is inadequate, secondary moments may appear, causing the construction to collapse.

**Figure 12:**
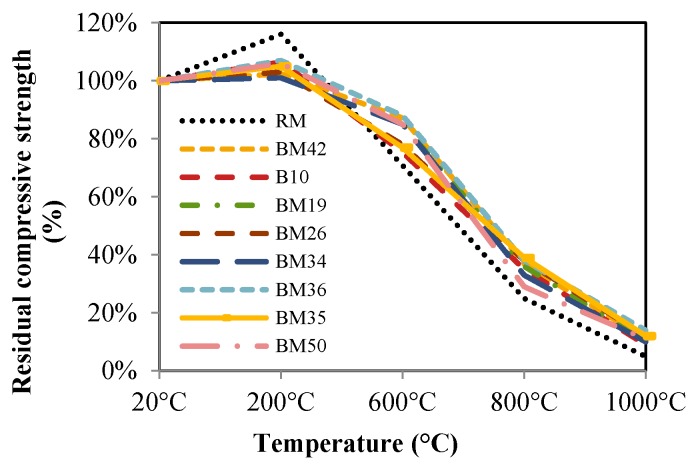
Residual compressive strengths.

Figure 12 shows the residual compressive strengths of concrete samples after they were exposed to high temperatures. Residual compressive strengths were calculated as a percentage of the remaining compressive strength after high temperature exposure, as compared to the initial compressive strength. The results in Figure 12 indicate that, after exposure to a temperature of 200 °C, the compressive strengths of all samples increased compared to the compressive strengths of samples cured at room temperature of 20 °C. The compressive strength increase compared to the initial values for the RM samples is 16%, and, for concrete containing CB and CT aggregates, the increase is between 2%–6%. According to [21,22,23], the compressive strength increase is caused by chemical changes in the cement paste. A loss of humidity occurs, due to which interparticle forces between hydration products increase, which subsequently leads to an increase in compressive strength. Another increase in compressive strength is presumably a shorter high temperature exposure for 200 °C compared to the duration of exposure at 600 °C, which also affects reason for the hydration reaction [22].

Concrete with CB and CT as aggregate displays a similar strength development profile at high temperatures as concrete with natural aggregate. The chemical changes of minerals for hardened cement paste and aggregates are dependent on the inner temperature of concrete.

Exposure of samples to 400 °C and higher temperatures cause a dehydration of calcium-hydroxide (CSH) in hardened cement paste. According to the results shown in Figure 12, it is possible to conclude that, up to approximately 400 °C, the decrease of the initial compressive strength of samples is small, and that the compressive strength loss is within a 10% limit. Presumably, the 10% decrease is the result of the elimination of free water and CSH dehydration from concrete [24,25].

After exposure of concrete to a temperature of 600 °C, the loss of compressive strength becomes more significant: for RM, it is 29%, and for CB and CT concrete, it is 13% to 25%. The samples with the highest percentages of CB and CT aggregate (BM36) show the lowest compressive strength loss at 600 °C, as expected. Crushed bricks and roof tile aggregates concrete enable a better passage of gas through concrete.

At temperatures higher than 800 °C, decarbonization of limestone occurs and the aggregate containing CaCO_3_ deteriorates, releasing CO_2_ as gas from the concrete. If the gas exit route is blocked, the resulting pressure causes microcracking and deterioration of the concrete structure. Since the brick and roof tile production process includes a basic limitation of the clay chemical composition, which is a maximum percentage of calcium-carbonate of 4%. CB and CT aggregates of concrete will presumably have a significantly better integration and higher remaining strength after high temperature exposure when compared to dolomite aggregate concrete, which contains around 30% calcium-carbonate. After exposure to high temperatures, the decarbonization of dolomite results in porous dolomite lime with a large specific surface area. The dolomite lime increases its volume under the influence of the earlier described reaction and gas leaves the concrete.

All samples exposed to 1000 °C show a low residual compressive strengths, e.g., RM 5%, BM36 11% and BM50 7%. The samples were tested immediately after cooling to room temperature, a day after they were heated. All samples that remained for seven days at room temperature, and humidity conditions deteriorated and fractures occurred as a result of dolomite expansion, as described in the previous section.

**Figure 13:**
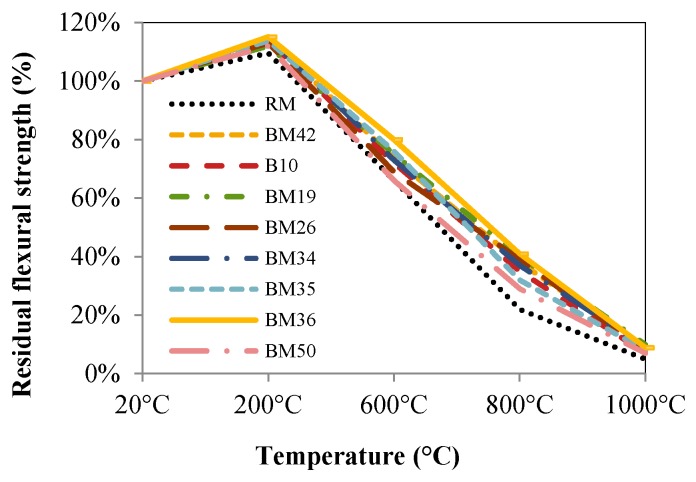
Residual flexural strengths.

CB and CT aggregates are sharper and coarse particles, which may result in a better bond between the aggregate particles and cement paste, subsequently resulting in better mechanical properties of concrete. The presented results indicate that CB and CT are better at tolerating high temperatures when compared to dolomite, due to the lower percentage of CaO in the chemical composition and the geometrical properties of its particles, as is evident in the percentage of the remaining compressive strengths illustrated in Figure 12. The residual flexural strengths are shown in Figure 13. All conclusions listed for residual compressive strengths of concretes RM to BM50 can be drawn also for residual flexural strengths. The samples of the BM36 mixture show the best properties with regard to residual flexural strengths.

**Figure 14:**
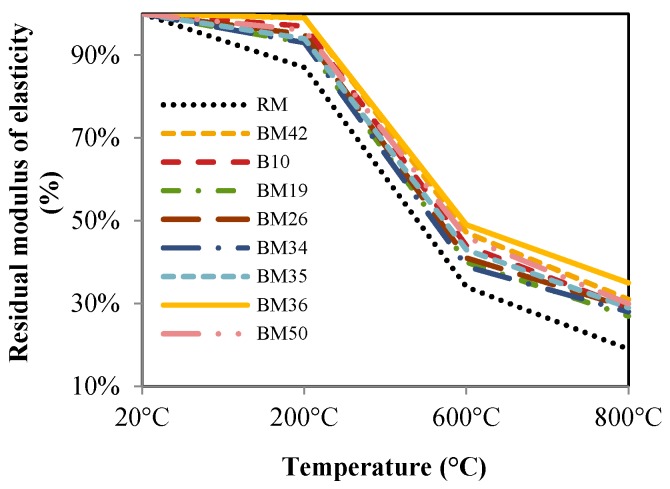
Residual modulus of elasticity.

**Figure 15:**
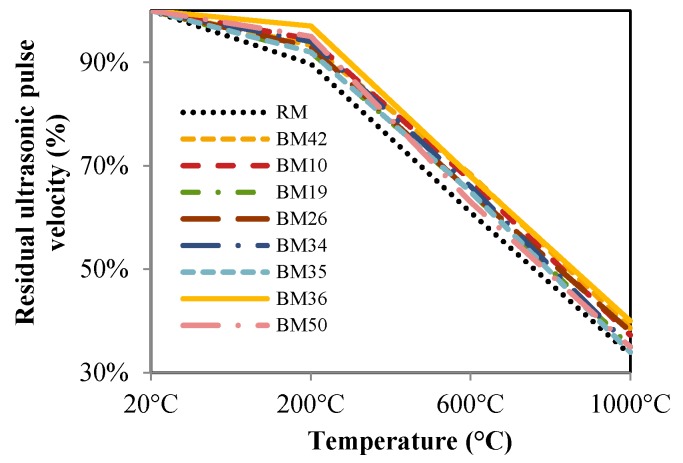
Residual ultrasonic pulse velocity.

The modulus of elasticity of concrete decreases after exposure to high temperatures, as is shown in Figure 14. Samples exposed to 1000 °C could not be tested because their outer surface peeled, making it impossible to properly position the specimen in the testing device and to attain proper results. Therefore, the tests were conducted only on samples exposed to 200, 600 and 800 °C. The results show that the residual modulus of elasticity of concretes with CB and CT as aggregates during exposure to high temperatures are continuously higher, about 10% to 13%. The highest residual modulus of elasticity after exposure to all temperatures was obtained for mixture BM36. By comparing Figure 12 and Figure 14, it can be seen that the reduction in modulus of elasticity obtained at increasing temperature is higher than the compressive strength reduction due to the deformation increase for the same strain value. Presumably, the decrease in deformation after exposure to high temperatures is due to the superior thermal properties of CB and CT compared to dolomite, which could imply the possibility of the application of such concrete in resisting or semi-resisting floor blocks. The ultrasonic pulse velocity through samples was determined before and after exposure to high temperatures, and the results are shown in Figure 15. Since the pore structure changes during the exposure of concrete to high temperature, this also affects mechanical properties. Measurement of the ultrasonic pulse velocity through samples after exposure to high temperatures was used to determine whether the appearance of cracks in concrete causes a reduction in mechanical characteristics. From Figure 15, it is evident that RM has an approximately 5% lower residual ultrasonic pulse velocity for all temperatures compared to mixtures with CB and CT aggregates, indicating that more cracks develop in concrete with NA.

**Figure 16:**
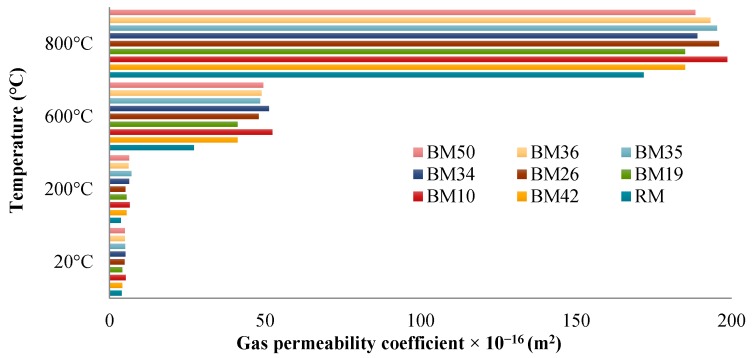
Gas permeability after exposure to high temperatures.

Considering that floor blocks will not be exposed to outside environmental conditions (exposure class X0), it is presumed that the permeability is the property with most pronounced with regard to durability. Since methods of laboratory testing of gas permeability have not been standardized yet but have been described and recommended by Technical Committee RILEM 116-PCD within the European organization RILEM [26], the tests were conducted in line with recommendations of Cembureau (RILEM TC 116–PCD). Figure 16 indicates that RM has a lower gas permeability coefficient than mixtures with CB and CT aggregates, making it the most suitable concrete with regard to gas permeability after high temperature exposure. It is also evident that all concrete mixtures fall into the category of poor-quality concretes with regard to gas permeability, and all earlier conclusions with regard to gas permeability tests at room temperature also apply for gas permeability in samples exposed to high temperatures. Figure 16 shows that the gas permeability is up to 47 times higher after exposure to temperatures above 600 °C than after exposure to a temperature of 200 °C. The reason for this is the appearance of cracks in the concrete. Surface cracks on samples of RM appear after the exposure to a temperature of 600 °C, and the number increases continuously at increasing temperature when they are exposed to 800 and 1000 °C. With all the other mixtures containing CB and CT aggregates, surface cracks appear only after the samples have been exposed to a temperature of 800 °C. Presumably, the cracks are smaller in size and fewer in number in samples with CB and CT due to the lower content of calcium-carbonate in these aggregates compared to dolomite.

**Figure 17:**
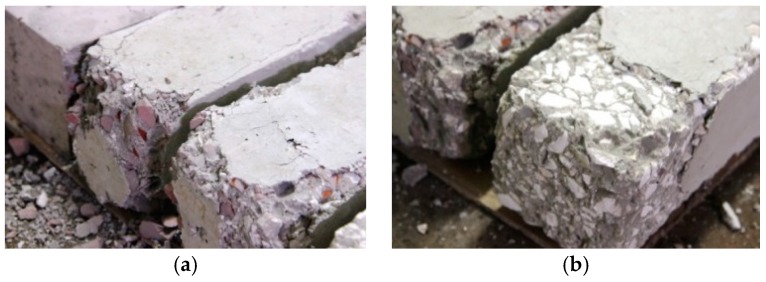
Examples of fire spalling of concrete surface after exposure to a temperature of 1000 °C for different concrete mixtures. (**a**) Concrete with CB and CT aggregates; (**b**) Mixture RM.

Figure 17a,b show samples seven days after their exposure to a temperature of 1000 °C. In Figure 17a,b shows spalling of the concrete surface above the aggregate particles in RM samples, whereas, in samples with CB and CT aggregates, the surface spalling occurs crossing the concrete matrix and dolomite particles. Brick and roof tile particles kept their integrity, underlining that CB and CT are appropriate for the production of fire-resistant concretes, as previously stated. Likewise, it may be claimed that the concrete matrix properties need to be improved with regard to its thermal properties. The use of highly-fire-resistant cement (for example, aluminium cement) or brick dust and roof tile dust as a partial cement substitute is recommended, which needs further research.

## 4. Conclusions

The review of possible applications for crushed clay bricks (CB) and roof tiles (CT) waste as partial substitution for natural aggregates (NA) in concrete identified the research area of this paper. The identification of research needs leads to the in depth research and proof of the possibility for using concrete with CB and CT as aggregates for the production of precast concrete elements.

The first step in the process of determining the properties of concrete with CB and CT as aggregates comprised experimental tests of CB and CT, which proved that such material may be used as a partial or full substitute for NA, depending on the required concrete properties.

Basic concrete properties were determined in order to optimize concrete with CB and CT as aggregates.

The densities of concrete mixtures with crushed brick and roof tiles (mixtures BM) are 11% to 14% lower than those of concrete with natural aggregate (mixture RM).

Experimental testing on BM and RM samples in the hardened state at room temperature showed that samples with CB and CT as aggregates compared to the reference samples RM had on average: a 27% lower compressive strength, a 16% lower flexural strength, a 19% lower hardened-state density, a 50% lower modulus of elasticity, a 20% lower ultrasonic pulse velocity, a 18% higher air permeability, and 28% less drying shrinkage.

It was concluded that special emphasis must be placed on using such concrete for horizontal and vertical structural elements (beams and columns) due to the lower modulus of elasticity and the possibility of larger deformations occurring.

The conducted laboratory experimental tests on mixtures BM with CB and CT aggregates after exposure to temperatures of 20, 200, 600, 800 and 1000 °C, and the comparison of the results with RM, lead to the conclusions:

- The BM36 mixture with the highest percentage of replacement of NA with CB and CT aggregates has the best physical, mechanical, and thermal properties with regard to the application of precast concrete elements exposed to high temperatures.
- In general, mixtures with CB and CT as aggregates have better physical and mechanical properties than regular concrete after exposure to high temperatures.

It is important to mention that this research is not applicable for crushed bricks and tiles in general (e.g., obtained from demolition of masonry structures), but for waste products of the process of making new bricks and roof tiles only. Furthermore, in order to make conclusions about the applicability of concrete with CB and CT aggregates in precast concrete elements in general (regardless of temperature to which the concrete is exposed), further research will be carried out to address the durability properties of the concrete.

## Figures and Tables

**Table 1:** Properties of CB, CT and NA determined according to [5].

Aggregate Type	CB		CT		NA	
	(Fine)	(Coarse *)	(Fine)	(Coarse *)	(Fine)	(Coarse *)
Particle shape (%)	-	7	-	11	-	6
Shape index	-	(SI15)	-	(SI15)	-	(SI15)
Particle size group	GF85	GC90/15	GF85	GC90/15	GF85	GC90/15
Specific density SSD (mg/m^3^)	2.13	2.16	2.29	2.25	2.86	2.88
Cleanliness (%)	4.4	0.4	3.6	0.8	3.2	0.8
Fines quality class	(f10)	(f1.5)	(f10)	(f1.5)	(f10)	(f1.5)
Los Angeles number/class	-	40 (LA30)	-	37 (LA30)	-	24 (LA25)
Water absorption (%)	19.05	16.71	11.50	9.25	2.29	0.50
Freezing/thaw resistance (%)	-	3.0	-	4.0	-	10.7
Magnesium sulphate class	-	(MS18)		(MS18)		(MS18)

* Coarse aggregate comprises fractions 4–8 mm and 8–16 mm.

**Table 2:** Concrete composition.

Mix Proportions (kg/m^3^)										
Code		RM	BM42	B10	BM19	BM26	BM34	BM35	BM36	BM50
Cement		300	300	360	400	300	400	400	400	400
Water		150	150	165.6	160	120	200	200	200	160
Water/Cement		0.50	0.50	0.46	0.40	0.40	0.50	0.50	0.50	0.40
Superplasticizer		-	-	1.08	2.00	1.50	-	-	-	-
Filler		208.17	208.17	197.68	-	-	-	-	-	-
Fine aggregate	CB	-	113.1	214.9	132.9	147.3	125.5	125.5	125.5	266.6
	CT	-	118.9	-	142.9	316.8	-	269.8	269.8	143.3
	NA	608.1	304.1	288.7	356.9	197.8	505.5	168.5	168.5	178.9
Coarse aggregate	CB	-	205.9	273.6	-	257.7	438.2	-	438.9	233.1
	CT	-	438.3	83.3	484.4	268.4	228.6	228.6	228.6	242.9
	NA	1170.9	292.8	611.6	620.0	687.2	292.6	877.9	292.6	621.7
Target strength (N/mm^2^)		40	25	30	20	20	25	30	40	40

**Table 3:** Properties of fresh concrete mixtures.

Code	RM	BM42	B10	BM19	BM26	BM34	BM35	BM36	BM50
Density (kg/m^3^)	2658	2296	2353	2149	2048	2088	2152	2185	2150
Slump (mm)	31.7	11.7	31.7	55.0	30.0	30.0	60.0	30	20
Slump class	S1	S1	S1	S2	S1	S1	S2	S1	S1
Air content (%)	2.5	4.4	4.3	5.0	4.6	5.4	4.8	4.4	5.5

**Table 4:** Properties of 28-day-old hardened concrete.

**Code**	RM	BM42	B10	BM19	BM26	BM34	BM35	BM36	BM50
Compressive strength (N/mm^2^)	39.36	25.53	31.95	22.24	20.53	24.02	34.2	41.72	42.67
Flexural strength (N/mm^2^)	5.71	4.34	5.31	5.68	4.72	5.07	5.32	5.79	5.88
Density of hardened concrete (kg/m^3^)	2479	2165	2250	2042	2005	2069	2147	2193	2125
Ultrasonic speed velocity (km/s)	5.03	3.89	4.14	3.52	3.46	3.77	4.79	5.12	5.43

**Table 5:** Properties of 56-day-old hardened concrete.

**Code**	RM	BM42	B10	BM19	BM26	BM34	BM35	BM36	BM50
Compressive strength (N/mm^2^)	45.33	33.42	37.33	23.32	21.33	24.43	34.80	42.24	44.80
Flexural strength (N/mm^2^)	6.03	4.96	5.38	5.73	4.91	5.22	5.48	5.81	5.92
Density of hardened concrete (kg/m^3^)	2476	2100	2184	1842	1839	1945	1978	1986	1973
Modulus of elasticity (MPa)	41.85	19.26	22.61	20.8	17.15	17.83	21.17	19.78	21.74
Ultrasonic speed velocity (km/s)	507	3.96	4.23	3.74	3,52	3.81	4.95	5.33	5.61
Gas permeability ×10^−16^ (m^2^)	3.49	4.10	5.25	3.84	3.72	3.96	3.57	3.61	3.56
